# Characteristics and outcomes of pulmonary barotrauma in patients with COVID-19 ARDS: A retrospective observational study

**DOI:** 10.5339/qmj.2024.64

**Published:** 2024-12-31

**Authors:** Varun Mahajan, Kamal Kajal, Amarjyoti Hazarika, Karan Singla, Naveen Naik B, Ananya Ray, Venkata Ganesh, Ajay Singh, Ashish Bhalla, Goverdhan Dutt Puri

**Affiliations:** 1 Department of Anaesthesia and Intensive Care, Postgraduate Institute of Medical Education and Research, Chandigarh, India; 2 Department of Anaesthesia and Intensive Care, BR Ambedkar Institute of Medical Sciences, Mohali, Punjab, India *Email: ananyaray1812@hotmail.com; 3 Department of Internal Medicine, Postgraduate Institute of Medical Education & Research, Chandigarh, India

**Keywords:** Coronavirus disease-2019, acute respiratory distress syndrome, pulmonary barotrauma, secondary bacterial infections, lower respiratory tract infection, patient-ventilator asynchrony, respiratory system compliance

## Abstract

**Introduction:**

Pulmonary barotrauma in coronavirus disease-2019 (COVID-19) acute respiratory distress syndrome (ARDS) carries high risk of mortality. While various studies have reported increased mortality, few have assessed the contributing factors for the occurrence of this complication. This study aimed at exploring the contributing factors for barotrauma in COVID-19 ARDS.

**Methodology:**

In this retrospective study, patients aged ≥18 years with laboratory confirmed severe acute respiratory syndrome coronavirus-2 (SARS-CoV-2) by reverse transcriptase polymerase chain reaction (RT-PCR) from a nasopharyngeal swab and having severe or critical COVID-19 disease requiring Intensive Care Unit (ICU) admission according to the World Health Organisation (WHO) criteria for disease severity in COVID-19 disease admitted at forty-bedded ICUs at a tertiary care research hospital in North India from April 1, 2020, to March 31, 2022 were included.

**Results:**

Of 825 patients admitted to COVID ICU, 40 developed pulmonary barotrauma, with a mortality rate of 85%. The mean ± SD PaO_2_/FiO_2_ was 96.76 ± 27.78 mmHg. Thirty-nine patients received steroids, 37 developed secondary bacterial infection of the lower respiratory tract with one or more organisms. *Acinetobacter baumannii* (*n* = 15), *Klebsiella pneumoniae* (*n* = 10), and *Pseudomonas aeruginosa* (*n* = 8) were the commonest isolates. Ten patients developed pneumomediastinum, of which 6 patients had subcutaneous emphysema along with pneumomediastinum, and 2 patients developed isolated subcutaneous emphysema. The remaining 28 patients developed pneumothorax.

The mean (±SD) for static respiratory system compliance (Crs) for patients on mechanical ventilation on the day of barotrauma was 19.3 (±10.5) mL/cmH_2_O.

**Conclusion:**

Patients with COVID-19 ARDS developing pulmonary barotrauma have a high associated mortality, and secondary bacterial infection, lung fragility, patient-ventilator asynchrony, as well as low respiratory system compliance, may contribute to lung injury, predisposing to barotrauma.

## 1. Introduction

The complication of barotrauma has often plagued mechanical ventilation of non-compliant lungs. The associated mortality, as high as over 75% in some reports, highlights the gravity of preventing this complication.^1^ It can present as pneumothorax, pneumomediastinum, pneumopericardium, subcutaneous emphysema, and/or tension pneumothorax. Of these, tension pneumothorax is the most dangerous as high intra-pleural pressures may restrict filling of the heart and great vessels, leading to hemodynamic collapse.

The concept of transpulmonary pressure (TPP) becomes contextual in the scenario of barotrauma. The TPP is the transmural pressure difference between the alveolus and the intrapleural space. Under normal circumstances, it amounts to 4 mmHg at rest as the intrapleural pressure is -4 mmHg and intra-alveolar pressure is 0 mmHg.^2^ This contributes to an outward pull and expansion of the lung tissue, thus causing the intra-alveolar pressure to be negative during inspiration and encouraging movement of air into the alveoli. Any TPP in excess of 60 mmHg due to increase in intra-alveolar pressure may set the stage for alveolar rupture and air escape into various extra-alveolar spaces.^3^ A schematic diagram of development of interstitial emphysema has been presented in Supplementary Figure 1. Hence, to prevent barotrauma, clinicians resort to techniques to minimize airway pressures and evade overdistension. The ARDSNet initiative developed various techniques to prevent volutrauma and barotrauma, including techniques for lung protective ventilation.^4^ This included reducing the tidal volume delivered to 6 mL/kg to avoid overdistension and maintain a plateau pressure of <30 cmH_2_O, which was found to significantly reduce mortality. Despite following protocols of lung protective ventilation, barotrauma continues to occur and contributes to increased morbidity and mortality. Although many reports exist describing this complication, the entire spectrum of contributing factors is not well understood.

The importance of this issue resurfaced during the coronavirus disease-2019 (COVID-19) pandemic which brought with it a wave of cases with acute respiratory distress syndrome (ARDS). In this pandemic, the incidence of barotrauma was found increased to up to 15% in patients with COVID-19 ARDS, as compared to those with non-COVID ARDS.^5^ Peculiarly, the COVID-19 pandemic also exhibited many cases with spontaneous barotrauma in non-mechanically ventilated patients. This highlighted the importance of self-inflicted lung injury in patients with respiratory distress. This begged us to raise the question as to what the various factors are that contribute to the occurrence of barotrauma. Hence, we aimed to estimate the factors associated with pulmonary barotrauma in COVID-19-positive patients admitted in the ICU, including the types of barotrauma, risk factors predisposing to barotrauma, associated secondary insults contributing to the development of barotrauma, and the outcomes of these patients.

### 1.1. Objectives

1. Estimate the incidence of barotrauma such as pneumothorax/pneumomediastinum/pneumopericardium and other types of barotrauma in patients requiring oxygen therapy in all three waves of the COVID-19 pandemic.
2. Describe the contributing factors for the development of barotrauma in COVID-19 ARDS.

### 1.2. Methodology

*Study type*: Retrospective cohort study

*Study setting*: Forty-bedded ICUs at a tertiary care research hospital in North India.

*Study duration*: April 1, 2020, to March 31, 2022.

*Study participants*: All patients meeting the following criteria:

*Inclusion criteria:*

- Patients aged ≥18 years.
- Laboratory confirmed severe acute respiratory coronavirus-2 (SARS-CoV-2) by reverse transcriptase polymerase chain reaction (RT-PCR) from a nasopharyngeal swab.
- Severe or critical COVID-19 disease requiring ICU admission according to the WHO criteria for disease severity in COVID-19 disease.^5^
- Patients requiring high-flow nasal cannula (HFNC), non-invasive ventilation (NIV), or invasive mechanical ventilation (IMV) for maintaining arterial partial pressure of oxygen >60 mmHg.
- Patients with chest X-ray findings of airspace opacities (consolidation and/or ground-glass opacities) consistent with COVID-19 disease.

*Exclusion criteria:*

- Patients admitted to ICU with incidental positive RT-PCR for COVID-19, not showing features of COVID-19 disease
- Iatrogenic or traumatic barotrauma, unrelated to COVID-19 disease.

*Data source*: Data were recorded from the daily patient electronic records. A separate database for COVID-19-positive patients who were admitted to a dedicated ICU had been maintained, from which data were extracted. The severity of the COVID-19 disease was defined according to the WHO criteria,^6^ and in patients with ARDS was defined according to the Berlin criteria^7^ and by the Kigali modification of the Berlin criteria.^8^

*Variables*: Data from patients who met the inclusion criteria were studied pertaining to the demographic characteristics, presenting symptoms, associated pre-existing lung disease such as bronchial asthma or chronic obstructive pulmonary disease (COPD), daily vital charts, ventilatory parameters, laboratory values, including culture reports, and radiological data. Chest radiographs had been performed at admission to hospital and daily thereafter. High-resolution computed tomography (HRCT) chest had not been done routinely for all the patients. The diagnosis of barotrauma in all cases was primarily based on chest radiographs. For patients receiving mechanical ventilation, ventilatory parameters had been set by the intensive care team based on ARDSNet recommendations targeting the minimum plateau pressure required to adequately achieve the minute ventilation demand of the patient to maintain a normal pH. Regular prone positioning sessions for 14–16 hours had been performed for patients with a PaO_2_/FiO_2_ ratio <100 mmHg. For data on respiratory system compliance in patients who were intubated post-barotrauma, the first reading of respiratory system compliance after initiating invasive mechanical ventilation has been considered for analysis.

*Primary endpoint*: The primary endpoint of the study was death or discharge from ICU in patients developing barotrauma during their ICU stay.

*Statistical analysis*: The primary endpoint is reported as mortality in percentage considering patients with barotrauma who expired while undergoing treatment in the ICU to the total number of patients who suffered barotrauma. Quantitative data are reported as absolute numbers and percentages. Continuous variables are summarized as means and standard deviation (SD) for normally distributed data and as median with interquartile range (IQR) for skewed data. Normality was assessed with the Shapiro-Wilk test and examination of the Q-Q plots. All statistics in our study are considered descriptive as the patient cohort was not selected at random. Normally distributed continuous data are reported as mean ± SD, and non-normally distributed continuous data are reported as median and IQR. All analyses were done using International Business Machines Corporation (IBM), Statistical Package for Social Sciences (SPSS). Statistics software for MacOS version 24 (Armonk, NY: IBM Corp).

*Ethics statement*: The study was conducted after approval of the Institute Ethics Committee (IEC), Reference number IEC/INT/2023/Study-1473. The need for the consent was waived off by the IEC due to the retrospective nature of the study.

*Guidelines for reporting*: This study adheres to strengthening of reporting of observational studies in epidemiology (STROBE) guidelines.

## 2. Results

**Figure 1:**
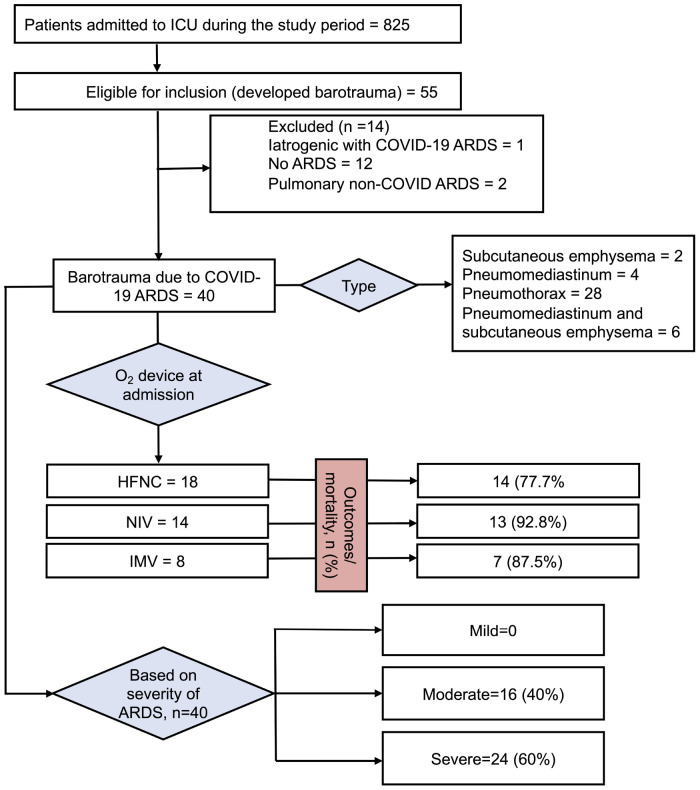
Study flow diagram.

**Figure 2:**
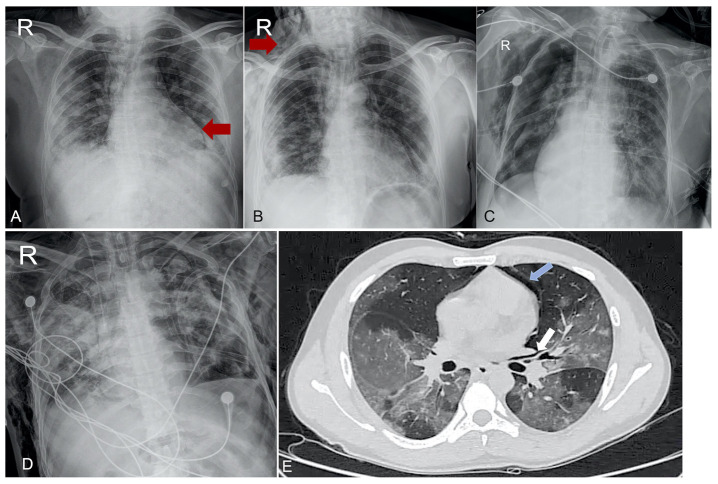
Radiographic characteristics of patients with COVID-19 ARDS. (A & B) A patient with COVID-19 ARDS developing spontaneous barotrauma beginning with pneumopericardium (A) and later progressing to subcutaneous emphysema (B). (C) A tracheostomized patient in the weaning mode developed bilateral pneumothorax requiring bilateral intercostal drain insertion. (D) A patient developed left-sided pneumothorax associated with bronchopleural fistula, for which two intercostal drains were inserted. The tracheal aspirate showed growth of Klebsiella pneumoniae. (E) High-resolution computed tomography of the chest showing peri-bronchial air (white solid arrow), indicative of positive Mackle’s sign and pneumopericardium (blue solid arrow).

A total of 825 patients were admitted to the ICU. Of these, 328 (39.7%) were females and 497 (60.3%) were males. The mean (±SD) age was 45 ± 17 years. Three hundred and fifty-two patients died, and 473 survived. In all, 55 patients admitted to ICU who developed barotrauma met the inclusion criteria initially. Of them, 14 were admitted for reasons other than COVID-19 disease and were incidentally diagnosed to have COVID-19 by RT-PCR for SARS-CoV-2, and one developed iatrogenic pneumothorax during central venous cannula insertion and hence were excluded (Figure 1). Of the 825 patients, 661 were admitted primarily for COVID-19 disease, and the rest 164 were incidentally detected to be SARS-CoV-2 by RT-PCR and did not have COVID-19 disease. Of the 661 patients with COVID-19 disease, 40 (6.05%) developed barotrauma. The patient demographic characteristics and symptomatology are presented in Table 1. The characteristics of barotrauma have been highlighted in Table 2 and in Figure 2.

Of the 40 patients who developed pulmonary barotrauma, 27 (67.5%) were males and 13 (32.5%) were females. The mean (±SD) age was 50 ± 14.6 years. The mean ± SD PaO_2_/FiO_2_ was 96.76 ± 27.78 mmHg. The commonest symptoms were dyspnea (100%), fever (90%), and cough (62.5%). Three patients had bronchial asthma, one had COPD, two had a history of pulmonary tuberculosis, and one patient gave the history of smoking.

Twenty-four patients had severe ARDS, mean ± (SD) PaO_2_/FiO_2_ of 77.8 ± 13.2 mmHg; sixteen patients had moderate ARDS, PaO_2_/FiO_2_ of 124.8 ± 17.2 mmHg; and none had mild ARDS. Of these 40 patients, 34 (85%) died during the ICU stay while the rest 6 (15%) were discharged to ward and later from the hospital. Twenty-five (62.5%) patients were on invasive mechanical ventilation at the time of barotrauma, 9 (22.5%) patients on NIV, and 6 (15%) on HFNC. Of the mechanically ventilated patients, 11 patients were on volume control modes, 10 on pressure control modes, while 4 were in weaning modes allowing spontaneous breaths. All patients were in supine position at the time of barotrauma.

Ten patients developed pneumomediastinum, of which 6 patients had subcutaneous emphysema along with pneumomediastinum, and 2 patients developed isolated subcutaneous emphysema. The remaining 28 patients developed pneumothorax. Of them, 19 patients had right pneumothorax, while 7 patients developed left-sided pneumothorax, and 2 patients developed bilateral pneumothorax. Five patients later developed bronchopleural fistula.

**Figure 3:**
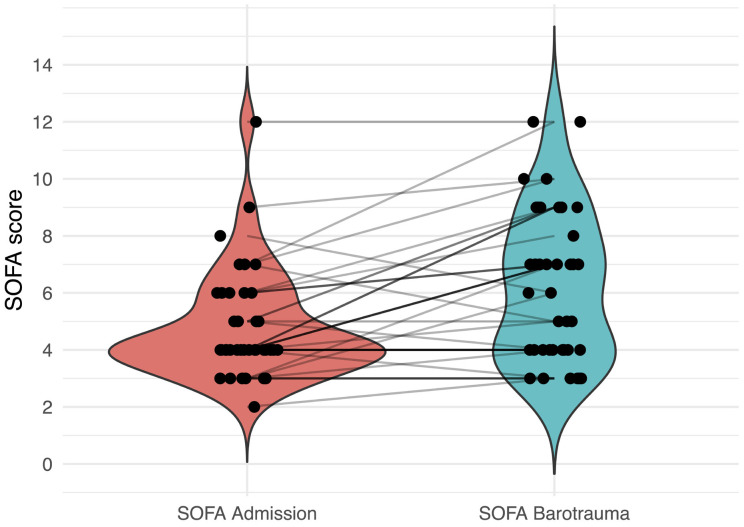
Violin plot representing the distribution of SOFA scores of the patients at admission and at the occurrence of barotrauma. The black dots represent the SOFA score of the subjects, and black lines are the trend lines showing the trend of SOFA score from admission to the occurrence of barotrauma. SOFA: sequential organ failure assessment score.

The mean (±SD) sequential organ failure assessment (SOFA) scores at admission and on the day of barotrauma were 5 ± 2 and 6 ± 3, respectively (Figure 3). The mean (±SD) for static respiratory system compliance (Crs) for patients on mechanical ventilation on the day of barotrauma was 19.3 (±10.5) mL/cmH_2_O, range 6.9–67 mL/cmH_2_O (*n* = 36). The median peak pressure of mechanically ventilated patients, recorded prior to barotrauma, was found to be 34.5 cmH_2_O (*n* = 25). Table 3 highlights the laboratory parameters at admission and at the time of barotrauma. The mean (±SD) PaO_2_/FiO_2_ ratio on the day of barotrauma was found to be 97.8 (±35.2) mmHg, and the median (IQR) PaO_2_/FiO_2_ ratio was 90.4 (69.95, 127.72) mmHg.

All but 5 patients received Remdesivir (87.5%), and 9 patients received Tocilizumab (22.5%) (Table 4). For initial steroid use, 26 (65%) patients had received injection dexamethasone prior to barotrauma, and 13 (32.5%) received injection methylprednisolone (Table 4). The steroid regimen used was injection dexamethasone 6 mg IV once daily for 10 days or injection methylprednisolone 40 mg twice daily for 5 days, which was followed by oral prednisolone 40 mg once daily according to the clinical condition of the patient and the clinicians’ decision. Injection remdesivir was administered at a dose of 200 mg twice a day for one day followed by once daily for further 4 days. Injection tocilizumab was administered at a dose of 400 mg IV single dose for patients weighing less than 60 kg, and 800 mg in 2 divided doses for patients weighing more than 60 kg. Injection enoxaparin was administered at a dose of 0.1 mg/kg twice a day, fondaparinux at 0.01 mg/kg once daily, and unfractionated heparin at 5000 IU thrice daily, all administered subcutaneously. Antiplatelets were not prescribed as a part of treatment protocol, but antiplatelet therapy for other comorbidities was continued with these medications.

Prophylactic antibiotics had been started for all patients with suspicion of community-acquired pneumonia on admission as a protocol, most commonly ceftriaxone and azithromycin, which were then changed depending on clinical deterioration and investigations suggestive of bacterial infection. This was further modified according to the laboratory confirmation of the isolates.

**Figure 4:**
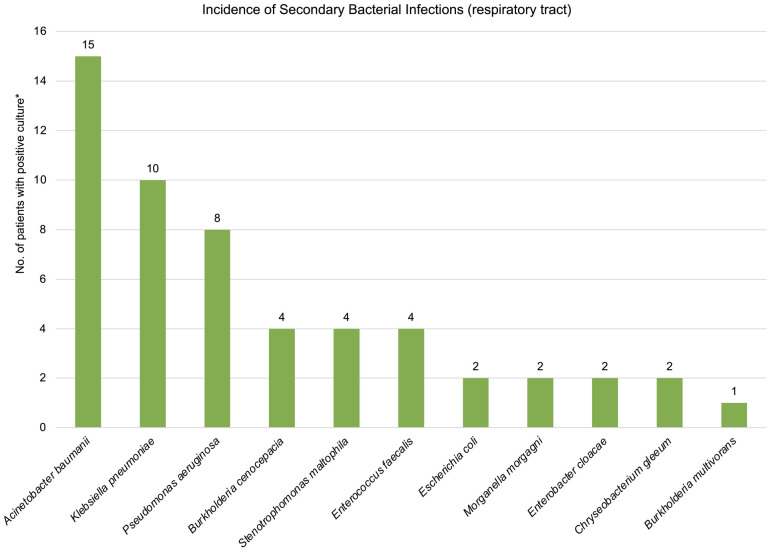
Bacterial isolates from the lower respiratory tract secretions in patients with severe COVID-19 admitted to ICU. *One patient could have more than one isolate at a given point in time. Twenty-seven (67.5%) patients had positive culture for lower respiratory tract organisms.

Twenty-seven (67.5%) patients had positive culture reports in the samples from the lower respiratory tract. Thirteen patients (32.5%) did not have any secondary bacterial infection of the respiratory tract. The commonest organisms isolated from respiratory cultures at the time of barotrauma were Acinetobacter baumannii (15), Klebsiella pneumoniae (10), and Pseudomonas aeruginosa (8) (Figure 4). Two patients had K. pneumoniae isolates which were resistant to polymyxins, and received a combination therapy with ceftazidime, avibactam, and aztreonam. Both of these patients died during the ICU stay. Eight patients (20%) had more than two isolates from the respiratory tract. No gram-positive isolates were found from the respiratory tract. Fourteen patients (35%) developed bloodstream infection during the ICU stay, most due to A. baumannii (4), K. pneumoniae (3), and P. aeruginosa (3). Three patients had concurrent fungal infection with yeast on culture, while 1 patient developed rhino-orbital mucormycosis. Seven other patients were administered anti-fungal therapy based on β-D-glucan and Galactomannan assays in addition to clinical suspicion. Two patients had positive potassium hydroxide (KOH) mount mount for septate hyphae in the lower respiratory tract secretions, and one of these patients had concurrent secondary bacterial infection of the lower respiratory tract with P. aeruginosa, Escherichia coli, and Enterococcus faecalis. Both the patients died during their ICU stay. One patient developed bloodstream infection with Candida tropicalis with concurrent secondary bacterial infection with Klebsiella pneumonia. This patient also died during the ICU stay.

Nineteen patients were receiving vasopressors on account of hemodynamic instability at the time of barotrauma, of whom 7 patients were on 2 vasopressors.

## 3. Discussion

In this retrospective analysis of patients with COVID-19 ARDS with barotrauma, we found a mortality of 85%, with most patients developing right pneumothorax. It was also associated with low lung compliance and secondary bacterial infection of the lower respiratory tract, most commonly with A. baumannii and K. pneumonia, and high serum inflammatory markers. The ventilatory mode (pressure vs. volume control), SOFA score trend from admission to incidence of barotrauma, and vasopressor administration were unlikely to contribute to barotrauma.

In a systematic review and meta-analysis, the incidence of barotrauma has been reported to be 4.2% (2.4–7.3%) among hospitalized patients, 15.6% (11–21.8%) in critically ill patients, and 18.4% (13–25.3%) in those requiring invasive mechanical ventilation.^9^ Another study involving 363 COVID-19 positive mechanically ventilated patients reported an incidence of 12%.^1^ Our figures find the incidence of barotrauma amongst critically ill COVID-19 patients to be somewhat lower at 6.05% with a mortality rate of 85%. The Steinberger et al., also reported a mortality of 77%, whereas the meta-analysis reported a mortality rate of 58.7%. Another multicentric study reported a mortality rate of 73% in their critically ill, mechanically ventilated patients.^10^ review article by Beletti et al. reported a mortality rate of 61.6% in mechanically ventilated COVID-19 patients who developed barotrauma.^11^ Similarly, we found a mortality rate of 85% in our cohort. The slightly higher rate may be attributed to the smaller sample size being considered in our study.

As in other studies,^5,12,13^ most of our cohort who developed barotrauma were on invasive mechanical ventilation (62.5%). We also had patients who developed barotrauma during spontaneous ventilation which is reported in only few studies.^13,14^ This highlights the importance of self-inflicted lung injury. Furthermore, our data showed that the incidence of barotrauma was similar with both volume control and pressure control ventilatory modes, suggesting that pressure limitation was not significant in preventing barotrauma. We also had patients who developed barotrauma on weaning modes of ventilatory support, suggesting a role of ventilator asynchrony contributing to lung injury. No study has reported regarding the significance of specific ventilatory modes so far. Nine patients were tracheostomized at the time of barotrauma and were being weaned off from the ventilator.

The commonest form of barotrauma in our patients was pneumothorax, especially right-sided pneumothorax. Most studies have reported right-sided pneumothorax as the most common manifestation of barotrauma as well.^5,15,16^ A study by Li et al. hypothesizes that COVID-19 preferentially targets the right lung as evidenced by computed tomographic studies of the pulmmonary lesions in COVID-19 ARDS.^17^ However, they too offer no explanation for this phenomenon and it remains a matter with potential for further study.

Our patients had very low Crs of the lung at the time of barotrauma, a characteristic not reported previously in studies. This led to a high mean peak pressure despite following ARDSNet ventilation strategies. Poor Crs necessitates ventilation at elevated peak and plateau pressures to achieve the desired minute ventilation, which increases predisposition to barotrauma. In our previously published study, we have shown that keeping the head end of bed at higher degree of elevation leads to overdistension of the functional alveoli and hence further reduction in the Crs.^18^ Hence, lower degrees of head end elevation (<30°) might help in reducing the alveolar overdistension whilst improving ventilation in patients requiring invasive mechanical ventilation (IMV). Similarly, prone ventilation in mechanically ventilated patients is another way to improve ventilation perfusion matching as confirmed recently by electrical impedance tomography studies.^19^ The lung mass increases in ARDS due to consolidation of the lung tissue. This leads to atelectasis of the dependent functional lung tissue and overdistension in the nondependent lung tissue.^20^ Although both these interventions might assist in improving ventilation, their effect on reducing barotrauma is not definitively known.

Most of our patients additionally had secondary bacterial infection of the lung. So far, only one study has addressed the presence of secondary bacterial infection in their cohort with barotrauma.^13^ In our study population, all the secondary bacterial infections (SBI) were due to gram negative bacteria, which is also supported by a previously performed study at our institute.^21^ None of the patients had gram-positive organisms isolated from the respiratory tract, a finding which is contrary to other studies done elsewhere in the world.^22,23^

Histological and functional alterations introduced by the viral infections, like cell loss, goblet cell dysfunction, reduced ciliary beat function and mucus clearance, and reduced oxygen exchange, are pathognomic of viral infection, which can contribute to secondary bacterial infections.^24,25^ SARS-CoV-2 can lead to cytokine induction, cellular dysfunction, and impaired innate and adaptive lymphocyte response.^26^ This can further be complicated with the use of immunosuppressants or immunomodulators, like steroids and tocilizumab, respectively.

Secondary bacterial infection of the lower respiratory tract leading to increased respiratory secretions leads to increased respiratory resistance and hence the need for repeated suctioning.^27^ While being ventilated with volume-controlled ventilation, increased airway resistance in such a case can lead to inadvertent increase in peak and plateau airway pressures,^28^ which, if ignored, can lead to barotrauma.^29^ On the other hand, in pressure control modes of IMV, hypoventilation due to low tidal volume delivery with increasing airway resistance can result in hypercarbia and subsequent respiratory acidosis and hemodynamic instability. End tidal carbon dioxide (EtCO2) monitoring with appropriately set alarms can help in preventing hypoventilation. In all the modes of IMV, peak pressure alarms should be appropriately set, and if the ventilator has a pressure-limiting option, it should be set appropriately.

Positive end-expiratory pressure (PEEP) is an essential component in the management of ARDS, and higher PEEP levels have been associated with early barotrauma in acute lung injury (ALI)/ARDS.^30^ Optimum PEEP is desirable to avoid barotrauma while avoiding the repeated collapsing of the alveoli.

The increased predisposition to developing barotrauma in COVID-19 ARDS could simply be due to increased frailty of the lung tissue, as advocated by Lemmers et al.^31^ In their study, despite the plateau pressures and PEEP used in their cohort, which were lower than the previously reported studies on non-COVID ARDS, the incidence of barotrauma was higher. Apart from this, there was no difference in the peak airway pressures, plateau pressures, and PEEP amongst those who developed barotrauma and those who did not.^31^

Most of the studies on barotrauma in COVID-19 ARDS have focused on the increased mortality in patients experiencing barotrauma.^1,5,15^ Rather, the risk factors for pulmonary barotrauma in COVID-19 ARDS should be explored to initiate timely targeted therapies for the factors associated with increased risk of pulmonary barotrauma in this patient population. Although the COVID-19 pandemic has subsided, future studies should focus on the inciting events in the development of barotrauma and factors which can prevent the development of barotrauma, as it is associated with a high mortality as depicted by previous studies.

## 4. Conclusion

Patients with COVID-19 ARDS developing pulmonary barotrauma might have a higher associated mortality, and secondary bacterial infection, lung fragility, patient-ventilator asynchrony, as well as low respiratory system compliance, may contribute to lung injury, predisposing to barotrauma.

## 5. Limitations

This study has limitations inherent to the retrospective studies. The true incidence of air leak may be higher than what was apparent as clinically detectable barotrauma reported in our study. Had it been a prospective study, the undetected subclinical BT could have been identified using the HRCT chest imaging, which was not done routinely at our hospital. Case-controlled comparison was not made in our study as this was an exploratory study into the possible etiologies of barotrauma in COVID-19 ARDS patients. Although we cannot conclude with certainty, strength of our study includes emphasis on the probable associations of barotrauma with severe cough, secondary bacterial infections of the respiratory tract, and patient-ventilator asynchrony during weaning as potential risk factors for barotrauma in these patients.

## Credit Authorship Statement

VM: Conceptualization, methodology, validation, formal analysis, investigation, resources, data curation, writing—original draft, approval of the final version, visualization, project administration. KK: Conceptualization, methodology, validation, investigation, formal analysis, data curation, writing—original draft, writing—review and editing, approval of the final version. AH: Conceptualization, methodology, validation, investigation, formal analysis, data curation, writing—original draft, writing—review and editing, approval of the final version. KS: Conceptualization, methodology, validation, writing—original draft, approval of the final version, supervision. BN: Conceptualization, methodology, validation, writing—original draft, approval of the final version, supervision. AR: Conceptualization, methodology, validation, formal analysis, investigation, resources, data curation, writing—original draft, approval of the final version, visualization, project administration. VG: Conceptualization, methodology, validation, writing—original draft, approval of the final version, supervision. AS: Conceptualization, methodology, validation, writing—original draft, approval of the final version, supervision. AB: Conceptualization, methodology, validation, writing—original draft, approval of the final version, supervision. GP: Conceptualization, methodology, validation, writing—original draft, approval of the final version, supervision.

## Supplementary Material

**Supplementary Figure 1:**
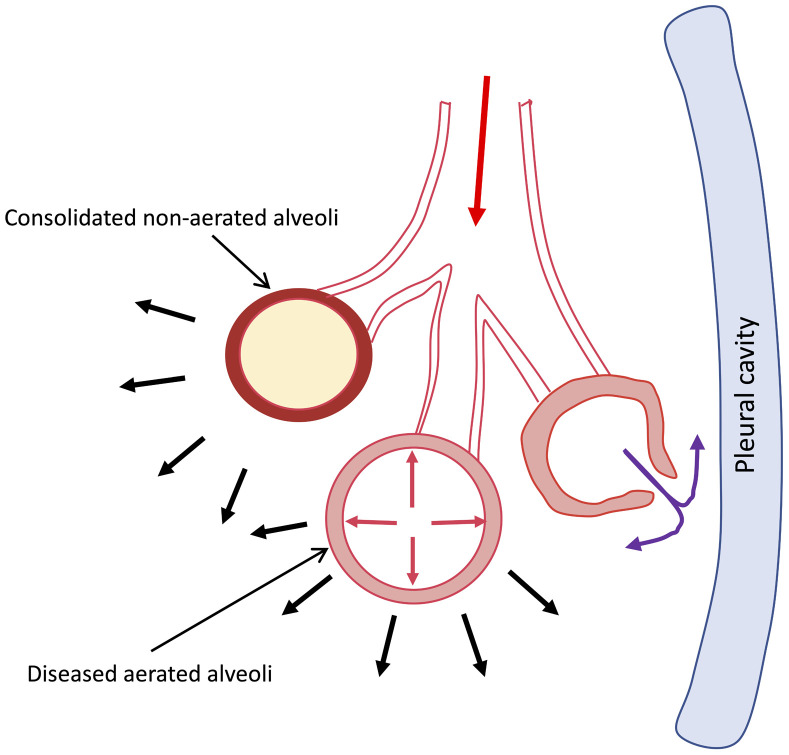
Schematic diagram to explain the pathophysiology of spontaneous barotrauma in severe COVID-19 disease. The overdistension of the diseased functional alveoli is contributed by increased inspiratory effort leading to increased negative trans-pulmonary pressure (depicted by black solid arrows) and coughing leading to increased intrathoracic pressure (depicted by red solid arrows). The overdistension of the diseased alveoli with poor compliance leads to air leak. The air leaks to the peribronchial tissue (purple solid arrows) manifesting as mediastinal emphysema or subcutaneous emphysema, which can further lead to pneumopericardium or pneumothorax when air leaks into the pericardial or pleural cavities, respectively. *Reference*: Macklin MT, Macklin CC. Malignant interstitial emphysema of the lungs and mediastinum as an important occult complication in many respiratory diseases and other conditions: An experimentation of the clinical literature in the light of laboratory experiment. *Medicine*. 1944;23(4):281–358.

## Figures and Tables

**Table 1.** Clinical characteristics of patients with barotrauma.

**Baseline vitals**	
SpO_2_, %	94 (91, 96)
Systolic blood pressure, mmHg	126 ± 37
Diastolic blood pressure, mmHg	74 ± 21
Mean blood pressure, mmHg	91 ± 25
Heart rate, beats per min	85 ± 25
2 patient were post cardiac arrest with ROSC	
**Oxygen device at admission to ICU**	
High flow nasal oxygen	6 (15%)
Non-invasive ventilation	9 (22.5%)
Invasive mechanical ventilation	25 (62.5%)
**Comorbidities^a^**	
Hypertension	22 (55%)
Type 2 Diabetes mellitus	11 (27.5%)
Chronic kidney disease	5 (12.5%)
Bronchial asthma	3 (7.5%)
Chronic obstructive pulmonary disease	1 (2.5%)
Coronary artery disease	4 (10%)
Cerebrovascular accident	1 (2.5%)
Congestive cardiac failure	1 (2.5%)
Seizure disorder	1 (2.5%)
Post renal transplant	2 (5%)
Rheumatic heart disease	1 (2.5%)
Past history of pulmonary tuberculosis	2 (5%)
Smoking history	1 (2.5%)
**Symptoms**	
Fever	36 (90%)
Cough	25 (62.5%)
Severe cough	5 (12.5%)
Expectoration	6 (15%)
Abdominal symptoms	2 (5%)
Dyspnea	40 (100%)
Hemoptysis	1 (2.5%)
Restrosternal chest pain	1 (2.5%)
PaO_2_/FiO_2_ (in mmHg) at admission	92.25 (76.85, 116.5)

SpO_2_: peripheral oxygen saturation; PaO_2_/FiO_2_: ratio of partial pressure of oxygen to fraction of inspired oxygen.

^a^One patient could have more than one comorbidities.

Numerical data presented as absolute numbers and percentages; continuous data presented as median (inter-quartile range).

**Table 2.** Characteristics of the barotrauma in COVID-19 patients.

**Timing of first diagnosis, *n* = 40**	
Before admission	1 (2.5%)
During hospital or ICU stay, before initiation of HFNC or NIV	14 (35%)
During ICU stay, after initiation of IMV	25 (62.5%)
**Type of initial barotrauma, *n* = 40**	
Subcutaneous emphysema	2 (5%)
Pneumomediastinum	4 (10%)
Pneumomediastinum and subcutaneous emphysema	6 (15%)
Pneumothorax	28 (70%)
**Location of the initial barotrauma, *n* = 40**	
Right	19 (47.5%)
Left	7 (17.5%)
Bilateral	14 (35%)
Days from symptom onset to barotrauma	19 (12.75, 24.25)
Days from hospital admission to barotrauma	10 (4.75, 16.25)
**Modality of first diagnosis, *n* = 40**	
Clinical examination (subcutaneous emphysema)	3 (7.5%)
Lung ultrasound and clinical examination	2 (5%)
Chest X-ray	35 (87.5%)
HRCT chest	0
**Complications/progression of the initial barotrauma, *n* = 40**	
Pneumomediastinum of subcutaneous emphysema to pneumothorax	3 (7.5%)
Pneumothorax to tension pneumothorax	6 (15%)
Bronchopleural fistula	5 (12.5%)
**Intervention needed, *n* = 40**	
Conservative	9 (22.5%)
ICD insertion unilateral	27 (67.5%)
ICD insertion bilateral	4 (10%)
**5 patients required needle thoracotomy as emergency procedure to relieve tension pneumothorax**	
Mortality based on initial diagnosis, *n* = 40	34 (85%)
Subcutaneous emphysema, *n* = 2	2 (100%)
Pneumomediastinum, *n* = 4	2 (50%)
Pneumomediastinum and subcutaneous emphysema, *n* = 6	4 (66.67%)
Pneumothorax, *n* = 28	26 (92.8%)
**Mode of oxygenation at barotrauma**	
HFNC	6 (15%)
NIV	9 (22.5%)
IMV	25 (62.5%)
**Nine patients were tracheostomised**	
PaO_2_/FiO_2_ (in mmHg) at diagnosis of barotrauma	90.4 (69.95, 127.72)
Duration of ICU stay, days	14 (10,22)
Duration of hospital stay, days	18 (12, 28)
Hospital admission to barotrauma, days	10 (4.75, 16.25)
Symptom onset to barotrauma, days	19 (12.75, 24.25)
**Change in mode of oxygenation after barotrauma**	
Already on IMV	24 (60%)
Required IMV after barotrauma	13 (32.5%)
Did not need IMV during ICU stay	3 (7.5%)

ICD: intercostal drain; ICU: intensive care unit; HFNC: high flow nasal oxygen; HRCT: high resolution computed tomography; IMV: invasive mechanical ventilation; NIV: non-invasive ventilation; PaO_2_/FiO_2_: ratio of arterial partial pressure of oxygen to fraction of inspired oxygen.

Numerical data presented as absolute numbers and percentages; continuous data presented as median (inter-quartile range).

**Table 3.** Laboratory investigations at admission and at barotrauma.

	**Admission to ICU**	**At barotrauma**
pH	7.38 ± 0.07	7.34 ± 0.08
PaO_2_, mmHg	72.38 ± 13.2	66.54 ± 12.25
	70.75 (64.15, 78.897)	66 (59.17, 73.45)
PaCO_2_, mmHg	38.7 ± 13.77	48.98 ± 14.63
HCO^3-^, mmol/L	22.3 ± 4.6	26.53 ± 6.8
Base excess, mmol/L	-2.36 ± 3.95	1.3 ± 6.42
	-1.75 (-5.2, 0.02)	-0.1 (-3.6, 5.77)
SaO_2_, %	93.18 ± 3.48	89.83 ± 7.91
FiO_2_, %	76.7 ± 16.8	74.52 ± 20.61
PaO_2_/FiO_2_, mmHg	96.76 ± 27.78	97.83 ± 35.21
Serum lactate, mmol/L	2.26 ± 1.15	2.14 ± 0.92
Hemoglobin, g/dL	12.1 (10.42, 3.4)	10.05 (9.05, 12)
Platelet count, ×10^3^/µL	199.5 (152.25, 293)	236.5 (150.75, 311.5)
Total leucocyte count, ×10^3^/µL	13.8 (9.92, 17.95)	16 (11.9, 19.25)
Neutrophil/lymphocyte ratio	16.4 (9.74, 30.45)	31.5 (14.12, 47.77)
C-reactive protein, mg/L	82.6 (49.05, 99.9)	56.78 (21.75, 97.45)
Serum ferritin, ng/mL	1084 (474, 1713)	996.5 (457.75, 1556)
D-dimer, ng/mL	1347.5 (583, 4839.75)	1322 (931, 1885)
Lactate dehydrogenase, U/L	698 (545, 850.5)	521.5 (401.25, 680.25)
Serum bilirubin, mg/dL	0.44 (0.32, 0.62)	0.49 (0.35, 0.75)
Serum creatinine, mg/dL	0.89 (0.69, 1.15)	0.83 (0.59, 1.2)
Urea, mg/dL	55.05 (41.57, 68.62)	60 (40.52, 118.72)

ICU: intensive care unit; HCO^3-^: serum bicarbonate; PaCO_2_: partial pressure of arterial carbon di-oxide; PaO_2_: partial pressure of arterial oxygen; FiO_2_: fraction of inspired oxygen; SaO_2_: arterial oxygen saturation.

Continuous data presented as mean (±SD) or median (inter-quartile range [IQR]).

**Table 4.** Immunosuppressive and anticoagulation therapy for COVID-19 disease.

**COVID-19 specific therapy**	
Steroids only	5 (12.5%)
Remdesivir + Steroids only	26 (65%)
Tocilizumab + Remdesivir + Steroids	9 (22.5%)
**Anticoagulation**	
Enoxaparin	32 (80%)
Fondaparinux once daily	2 (5%)
Unfractionated heparin	6 (15%)
**Antiplatelets^a^**	
Aspirin	5 (12.5%)

COVID-19: coronavirus disease-2019.

Data presented as absolute numbers and percentages.

The steroids used were injection dexamethasone 6 mg IV once daily for 10 days or injection methylprednisolone 40 mg twice daily for 5 days, which was followed by oral prednisolone 40 mg once daily according to the clinical condition of the patient and the clinicians’ decision. Injection remdesivir was administered at a dose of 200 mg twice a day for 1 day followed by once daily for further 4 days. Injection tocilizumab was administered at a dose of 400 mg IV single dose for patients weighing less than 60 kg, and 800 mg in 2 divided doses for patients weighing more than 60 kg. Injection enoxaparin was administered at a dose of 0.1 mg/kg twice a day, fondaparinux at 0.01 mg/kg once daily, and unfractionated heparin at 5000 IU thrice daily, all administered subcutaneously.

^a^Antiplatelets were not prescribed as a part of treatment protocol, but those antiplatelet therapy for other comorbidities were continues with these medications.
